# Supplementary material for: Effects of Chinese traditional ethnic sports on sleep quality among the elderly: a systematic review and meta-analysis
Source: PeerJ. 2025 Feb 21;13:e19019. doi: 10.7717/peerj.19019 (PMC11849518; doi:10.7717/peerj.19019)
Supplement: Supplemental Information 3 [file peerj-13-19019-s003.docx]

# Appendix

WOS retrieval mode:

((((((((TS=(Qigong)) OR TS=(Mind Body exercise)) OR TS=(Fitness Qigong)) OR TS=(Traditional Chinese Exercise)) OR TS=(tai chi)) OR TS=(taiji)) OR TS=(baduanjin)) OR TS=(wuqinxi)) OR TS=(shierduanjin) AND (((TS=(sleep quality)) OR TS=(sleep)) OR TS=(disorders of initiating and maintaining sleep)) OR TS=(insomnia) AND ((((TS=(The aged)) OR TS=(old age)) OR TS=(senior citizen)) OR TS=(Elderly)) OR TS=(Aged) AND ((((TS=(randomized controlled trial)) OR TS=(randomized)) OR TS=(controlled)) OR TS=(trial)) OR TS=(RCT)

Pubmed Search method:

((((("Qigong"[Mesh]) OR ("Tai Ji"[Mesh])) OR ((((((((("Qigong"[Title/Abstract]) OR ("Mind Body exercise"[Title/Abstract])) OR ("Fitness Qigong"[Title/Abstract])) OR ("Traditional Chinese Exercise"[Title/Abstract])) OR ("tai chi"[Title/Abstract])) OR ("taiji"[Title/Abstract])) OR ("baduanjin"[Title/Abstract])) OR ("wuqinxi"[Title/Abstract])) OR ("shierduanjin"[Title/Abstract]))) AND ((("Sleep Quality"[Mesh]) OR ("Sleep"[Mesh])) OR (((("sleep quality"[Title/Abstract]) OR ("sleep"[Title/Abstract])) OR ("disorders of initiating and maintaining sleep"[Title/Abstract])) OR ("insomnia"[Title/Abstract])))) AND (("randomized controlled trial"[Publication Type]) OR ((((("randomized controlled trial"[Title/Abstract]) OR ("randomized"[Title/Abstract])) OR ("controlled"[Title/Abstract])) OR ("trial"[Title/Abstract])) OR ("RCT"[Title/Abstract])))) AND (("Aged"[Mesh]) OR ((((("The aged"[Title/Abstract]) OR ("old age"[Title/Abstract])) OR ("senior citizen"[Title/Abstract])) OR ("Elderly"[Title/Abstract])) OR ("aged"[Title/Abstract])))

Embase Search method:

('aged'/exp OR 'the aged':ti,ab OR 'old age':ti,ab OR 'senior citizen':ti,ab OR elderly:ti,ab) AND ('Qigong'/exp OR 'tai chi'/exp OR 'mind-body exercise':ti, ab OR 'Fitness Qigong':ti, ab OR 'traditional Chinese exercise':ti, ab OR taiji: ti, ab OR Baduanjin: ti, ab OR wuqinxi: ti, ab OR shierduanjin: ti, ab) AND ('sleep quality'/exp OR 'insomnia'/exp OR 'sleep'/exp OR 'disorders of initiating and maintaining sleep':ti, ab) AND ('randomized controlled trial'/exp OR randomized: ti, ab OR controlled: ti, ab OR trial: ti, ab OR rct: ti, ab)

Cochrane Search method:

#1 MeSH descriptor: [Qigong] explode all tree

#2 MeSH descriptor: [Tai Ji] explode all trees

#3 (“Qigong” or "Mind Body exercise" or "Fitness Qigong" or "Traditional Chinese Exercise" or "Tai chi" or "Taiji" or "baduanjin" or "wuqinxi" or "shierduanjin" Qigong or "Mind Body exercise" or "Fitness Qigong" or "Traditional Chinese Exercise" or "tai chi" or "taiji" or "baduanjin" or "wuqinxi" or "shierduanjin")： ti, ab,kW (Word variations have been searched)

#4 #1 or #2 or #3

#5 MeSH descriptor: [Sleep Quality] explode all trees

#6 MeSH descriptor: [Sleep] explode all trees

#7 (“sleep quality” or "sleep" or "disorders of initiating and maintaining sleep" or "insomnia")： ti, ab,kW (Word variations have been searched)

#8 #5 or #6 or #7

#9 MeSH descriptor: [Sleep] explode all trees

CNKI retrieval method:

Theme: (yi jin jing + wuqinxi + six-word formula + Fitness Qigong eight jin + 12 paragraph jin + fitness Qigong, big dance, fitness Qigong, Qigong 12 method, tai chi health staff + +, tai chi + tai chi + Qigong) AND theme: (sleep quality, life quality, sleep, sleep disorders, sleep phenomenon + insomnia) AND, theme: (older adult, the elderly, the elderly)

Wanfang Search method:

Title or keywords: (jin or birds play or six-word formula or fitness Qigong eight jin or 12 jin or fitness Qigong, big dance or fitness Qigong, guide health method or Qigong, tai chi health staff or health or tai chi or tai chi) and title or keywords: (sleep quality or life quality or sleep disorder or sleep phenomenon or insomnia) and title or keywords: (older man or elderly or older adults)

The VIP retrieval method:

Title or keywords: (easy jin classics or five birds or six-word formula or fitness Qigong eight jin or 12 jin or fitness Qigong, big dance or fitness Qigong, guide health method or Fitness Qigong, tai chi health staff or tai chi or Qigong) and title or keywords: (sleep quality or life quality or sleep disorder or sleep phenomenon or insomnia) and title or keywords: (older adult or elderly or older adults elderly)
